# Continued Fever, or a So-Called Ridge Fever, in Upper South Carolina

**Published:** 1901-10

**Authors:** J. H. Moore

**Affiliations:** Fair Play, S. C.


### CONTINUED FEVER, OR A SO-CALLED RIDGE FEVER, IN UPPER SOUTH CAROLINA.*

BY. J. H. MOORE, M.D.,
FAIR PLAY, S. C.

*Mr. President and Gentlemen of the Oconee County Medical Society:*

In assuming the task you have imposed upon me, I do so with reluctance, as I had expected that the older members of our society would be called upon to lead the discussion, while I would have been content to occupy the humble position of listener, drinking in the words of wisdom as they fell from the lips of experience, and appropriating truths gathered during years of practice in our noble profession. Then, too, gentlemen, I have been somewhat at a loss for a subject for this paper, not wishing to tread on ground already covered at our previous meetings. However, as the season for *continued fever* is still with us, and as the subject has not been disposed of to my entire satisfaction, I have presumed to discuss it again, trusting that it may not be unprofitable. At our meeting in May the paper which was read on "continued fever," put me to studying the subject as I had never studied it before. As you remember, it described a peculiar form of continued fever, which was designated by various names, but more particularly as "*ridge fever*," and the majority of the members present at that time seemed to agree that this particular fever was not typhoid, typho-malaria, nor malaria, but a different fever altogether; not described in the text-books, but a disease often producing death, and hence to be dreaded. At that time, I, representing a very small minority, took the ground that it was only an *atypical* form of typhoid fever, and since that time, after studying all the authorities at my command, and being called upon to treat several cases of such fever, I have found no reason to change my opinion as then expressed; and now I shall proceed briefly to give a few reasons for the faith that is in me.

*Read before Oconee County Medical Society, Westminster, S. C., September 4, 1901.

First, I will notice that the so-called *ridge fever* usually appears about June 1st, and rarely after January 1st, following, and most cases seem to originate after protracted periods of high temperature and little moisture.

Osler, in his practice, 2nd edition, page 3, in discussing typhoid fever, says:

"It prevails most in autumn months, and has been well called the autumnal fever, and has been observed to prevail most in hot and dry seasons. According to Pettenkofer, epidemics are most common when the ground water is low, under which circumstances the springs and water sources drain more thoroughly contaminated foci, and we are more likely to be highly charged with poison. It may be also, as Baumgerten suggests, that in dry seasons the poison is more disseminated in the dust."

In Sajous, An. and Analytical Cyclopedia of Practical Medicine, Vol. 6, page 567, I find the following in reference to typhoid fever:

"The great majority of cases occur during the last four months of the year, and from February to May there is comparative freedom from the disease. It is especially prevalent after hot, dry seasons, and less so when the summer has been wet and cold."

Loomis, in 8th edition, page 663, says:

"Typhoid fever frequently occurs in the same locality year after year, when the surrounding conditions are favorable to its development. Those conditionsare more frequently present in the autumn than any other season of the year, and for this reason it has been called autumnal fever."

Certainly, from these authorities, we could make no distinction between the so-called *ridge fever* and *typhoid fever* as to the seasons of its prevalence.

Now, as to the symptoms of the so-called *ridge fever* (which I wish to compare with those of typhoid), I will first notice the prodromal, which are said to be few, and substantially as follows:

Headache most prominent, tired feeling for few days to a week, amorixia, muscular pains in back and extremities, and when first seen by the doctor, the patient has a temperature of 102° to 104°, with occasional rigors, pulse 110 to 115, tongue coated white, constipated *nearly* always, etc. Now, with the *possible* exception of the constipation, I certainly would expect to find all of these symptoms in any well-marked case of typhoid fever. In the second week of the disease, it is claimed that the tongue assumes a brown coating, the temperature not varying so much morning and evening, and possibly some abdominal tenderness, with constipation still present. The third week finds patient emaciated, tongue beginning to clear at point and edges, temperature dropping lower, and by 21st to 28th day patient is convalescent.

I fail to see anything in the above symptoms and course of the disease that is not pathognomonic of typhoid fever, with the exception of constipation, and I have learned from experience to expect that condition as often as I do the opposite.

In regard to the negative symptoms, I note in the beginning that it is not claimed that constipation is *always* present in *ridge fever*, but *nearly always.*

In Sajous. An. and Analyt. Cyclopedia, vol. 6, page 552, under head of typhoid fever, I find the following:

"The patient complains of a general feeling of weariness, of loss of appetite, slight nausea, and *sometimes* of diarrhea, which is increased by mild cathartics." The writer here certainly leaves a doubt in the mind of the reader as to whether diarrhea is present in *all* cases. Osler, second edition, page 11, says:

"The bowels *may be constipated*, or there may be two or three loose movements daily."

Loomis, eighth edition, page 666, says: "Although not *invariably* present, diarrhea is so frequent an attendant of this fever that it is considered one of its characteristic symptoms. It varies with the commencement of the attack, the date of its commencement and its duration. In mild cases diarrhea is sometimes absent."

From the above, I certainly would not feel justified in making the statement that constipation never existed in typhoid fever.

As to the erratic temperature of the so-called *ridge fever*, Osler, on page 13, speaking of typhoid fever, says: "Variations in the normal temperature curve are *common*. We do not always see the gradual step-like ascent in the early stages; the cases do not often come under observation at this time. When the disease sets in with a chill, the temperature may rise at once to 103° or 104°. In *many* cases deferescence occurs at the end of the second week, and the temperature may fall rapidly, reaching normal within twelve to twenty hours. An inverse type of temperature, *high in the morning* and *low* in the evening, is occasionally seen, but has no especial significance."

Also, on page 15, he says: "During convalescence, after temperature has been normal perhaps for five or six days, the fever may rise suddenly to 102° or 103°, and after persisting for from one to three days, or even longer, falls to normal. With this there is no constitutional disturbance, no furring of the tongue, no distention of the abdomen. These so-called recrudescences are by no means uncommon, and are of special importance, as they cause great anxiety to the practitioner. They are attributed most frequently to errors in diet, *constipation*, emotions, and excitement of any sort, such as seeing friends. There are cases in which the temperature declines almost to the normal at the end of the third week, the tongue cleans and the patient enters apparently upon a satisfactory convalescence. The evening temperature does not reach 98.5, but constantly keeps about 99.5, or 100, and occasionally rises to 100.5. This, in the late stages of convalescence, I have seen due to the post-typhoid æuemia. In certain of these cases, the persistence of the fever seems to be really a nervous phenomenon, and there is nothing in the condition of the patient to cause uneasiness, except the evening elevation of temperature."

Sajous, vol. 6, page 559, says: "Cases of apyretic typhoid have from time to time been reported in which the temperature has never risen above the normal standard, and in some there has been a decline as low as 95°. Also, cases occasionally occur in which the temperature in the fourth, fifth or even sixth week pursues a most irregular course. They are often marked with rigors, with rapid elevations, sometimes as high as 105° or 106°, followed by equally rapid descents, often to below normal."

Loomis, eighth edition, page 664, says: "This gradual rise of, and these variations in temperature, *are not present in every case*, but when they are present, they will greatly assist in making an early diagnosis."

Also Osler, on page 1, in defining typhoid fever, says: "Clinically the disease is marked by fever, a rose-colored eruption, diarrhea, abdominal tenderness, tympanitis and enlargement of the spleen, *but these symptoms are extremely inconstant, and even the fever varies in its characters.*" Also, on page 8, the same authority says: "Are the enteric lesions constant and specific? In an immense majority of all cases the intestinal lymph follicles are involved, but it is claimed that exceptionally the disease may exist without lesions of the bowel. Several instances are reported, but DuCazels's case is the most satisfactory. The symptoms were those of typhoid fever, and at the autopsy the spleen, mesenteric glands and kidneys were swollen and congested. *There was no lesion of the intestine.* Typhoid bacilli were isolated by the most approved recent methods."

Sajous, vol. 6, page 551, in defining typhoid fever, says: "It must be remembered that fatal cases occur in which the intestines are quite normal and the lesions are found in other parts of the body."

The same author names no less than ten different varieties of typhoid fever, and hence it must follow that there are as many different sets of symptoms in order to make the distinctions.

In speaking of the diagnosis of typhoid fever, Dr. Osler, in *N. Y. Med. Jour.*, Nov. 4, '99, says: "There is no one symptom, there are no two or three symptoms, which in themselves are characteristic of the disease. There is no one symptom, there are no two or three symptoms usually occurring in the disease, *which may not be absent during its entire progress.*"

According to the above authorities we would hardly expect to find *all* the characteristic symptoms that usually accompany typhoid fever present in *all* cases.

As to the "Widal test," which is said to be negative in the so-called *ridge fever,* I will quote from Wm. H. Welch, in *Jour. Am. Med. Ass.*, August 14, '97. He says: "As the reaction may be delayed, or occasionally absent, a negative result of the test *does not exclude the diagnosis of typhoid fever.*"

In the *Monthly Cyclopedia*, February, 1900, page 51, I find the following: "A negative result from the 'Widal test' *cannot be regarded as having much significance*, but a positive reaction may be looked upon as almost conclusive proof of the presence of typhoid fever."

The inference here seems to be that, with a positive reaction from the "Widal test," you are very certain to have typhoid fever, *but with a negative result you are not certain that you have not got typhoid fever!*

So summing up the foregoing and finding so many of the symptoms present in the so-called *ridge fever* that we *know* to exist in typhoid, I am forced to the conclusion that *they are one and the same disease*, and should be so regarded.

That it is not of malarial origin I think is proven by the fact that quinine, however judiciously administered, has no control over the fever whatever.

I have quoted somewhat liberally in order to prove my claim that the text-books *do describe these a typical form of typhoid fever*, by teaching us not to expect to find all the prominent symptoms of typhoid fever in all cases, but rather to expect some, or many of them to be absent in any case.

As regards the treatment, I will only outline the one I employ, briefly: I usually give quinine in large doses for first twenty-four to forty-eight hours, unless I am satisfied of my diagnosis, when it is discarded. I give aro. sulph. acid gtts. x. every four hours, sulpho carbolate zinc (or soda) grs. iii. to v. every three hours. Bin. iodine mercury $\frac{1}{30}$ gr. doses three to four times daily, relieving constipation with sulphate magnesia. Control fever by sponging, liquid diet, and treat special symptoms as they arise.

Without making any egotistical claims, I will say that the above treatment has proven very satisfactory in my hands, the disease rarely lasting over two weeks, often temperature reaches normal in ten to twelve days, and the patient convalesces satisfactorily.

In conclusion I wish to thank Dr. Hines, of Seneca, for a copy of his paper, read before this society in May, which has been a great help to me in preparing this paper.

